# Persistence with Anticoagulation for Atrial Fibrillation: Report from the GLORIA-AF Phase III 1-Year Follow-up

**DOI:** 10.3390/jcm9061969

**Published:** 2020-06-23

**Authors:** Monika Kozieł, Michał Mazurek, Christine Teutsch, Hans-Christoph Diener, Sergio J. Dubner, Jonathan L. Halperin, Chang-Sheng Ma, Kenneth J. Rothman, Axel Brandes, Miney Paquette, Kristina Zint, Lionel Riou França, Shihai Lu, Dorothee B. Bartels, Menno V. Huisman, Gregory Y. H. Lip

**Affiliations:** 1 Liverpool Centre for Cardiovascular Science, University of Liverpool and Liverpool Heart & Chest Hospital, Liverpool L7 8TX, UK; kozielmonika@poczta.fm; 2 1st Department of Cardiology and Angiology, Silesian Centre for Heart Diseases, 41-800 Zabrze, Poland; m.i.c.h.a.l@wp.pl; 3 Department of Clinical Development and Medical Affairs, Therapeutic Area Cardiometabolism, Boehringer Ingelheim International GmbH, 55216 Ingelheim am Rhein, Germany; christine.teutsch@boehringer-ingelheim.com; 4 Institute for Medical Informatics, Biometry and Epidemiology, University of Duisburg-Essen, 45133 Essen, Germany; hans.diener@uk-essen.de; 5 Clínica y Maternidad Suizo Argentina, Buenos Aires C1420, Argentina; dubnermd@gmail.com; 6 Icahn School of Medicine at Mount Sinai, New York, NY 10001, USA; jonathan.halperin@mountsinai.org; 7 Cardiology Department, Atrial Fibrillation Center, Beijing AnZhen Hospital, Capital Medical University, Beijing 100011, China; chshma@vip.sina.com; 8 RTI Health Solutions, Research Triangle Park, NC 27709, USA; krothman@rti.org; 9 Department of Cardiology, Odense University Hospital, 5000 Odense, Denmark; axel.brandes@rsyd.dk; 10 Department of Medicine, Boehringer Ingelheim, Burlington, ON 05401, Canada; Miney.paquette@boehringer-ingelheim.com; 11 Global Epidemiology Department, Boehringer Ingelheim International GmbH, 55216 Ingelheim am Rhein, Germany; kristina.zint@boehringer-ingelheim.com (K.Z.); leonel@worldonline.fr (L.R.F.); bartels.dorothee@mh-hannover.de (D.B.B.); 12 Sanofi-Aventis Recherche et Development, 91380 Chilly-Mazarin, France; 13 Biostatistics and Data Sciences Department, Boehringer Ingelheim Pharmaceuticals, Inc., Ridgefield, CT 06877, USA; shihai.lu@boehringer-ingelheim.com; 14 Hannover Medical School, 30159 Hannover, Germany; 15 Department of Thrombosis and Hemostasis, Leiden University Medical Center, 1043 AJ Leiden, The Netherlands; m.v.huisman@lumc.nl; 16 Aalborg Thrombosis Research Unit, Department of Clinical Medicine, Aalborg University, 9000 Aalborg, Denmark

**Keywords:** atrial fibrillation, dosing frequency, GLORIA-AF, non-vitamin K antagonist oral anticoagulants, oral anticoagulants, vitamin K antagonists

## Abstract

Background: We aimed to assess the extent to which drug persistence is better with non-vitamin K antagonist oral anticoagulants (NOACs) than vitamin K antagonists (VKAs) in atrial fibrillation (AF) patients and to estimate the difference in therapy persistence depending on NOAC dosing regimen (once daily (QD) vs. twice daily (BID)). Methods: Consecutive patients were followed for 1 year in phase III of the GLORIA-AF registry. Drug persistence was defined as the use of OAC without any discontinuation in >30 days or switching to alternative therapy. Results: Among 21,109 eligible patients in phase III, 17,266 patients who were prescribed OAC at baseline and those who took ≥1 OAC dose were included. The 1-year proportion of patients receiving NOAC and VKA who persisted on treatment was 80% and 75%, respectively. The 1-year persistence with NOACs BID and NOACs QD was 81% and 80%, respectively. Female gender, hypertension, older age, alcohol use, permanent, asymptomatic, and minimally symptomatic AF were associated with better OAC persistence. Region, medication usage predisposing to bleeding, being a current smoker, treatment reimbursement, and proton pump inhibitors were associated with lower OAC persistence. Conclusions: Drug persistence was higher with NOACs (1-year persistence was 80%) than with VKAs (75%). There was little difference in 1-year persistence between NOAC dosing regimens.

## 1. Introduction

Atrial fibrillation (AF) is the most prevalent cardiac arrhythmia. It is associated with an increased risk of ischaemic stroke, stroke-related morbidity, and mortality [1]. Oral anticoagulant (OAC) therapy is recommended to prevent thromboembolism in patients with AF with ≥1 stroke risk factors [2]. The non-vitamin K antagonist oral anticoagulants (NOACs) have an improved efficacy/safety ratio when compared with vitamin K antagonists (VKAs), and are recommended as first-line therapy for stroke prevention in AF [2].

Furthermore, the use of VKA may be limited due to its narrow therapeutic interval, fluctuations of dose–response relationship, and interactions with drugs for concomitant diseases and with food [3]. However, therapy persistence with an OAC is crucial for the maintenance of effective and optimal stroke prevention over time, and several reports suggest that therapy persistence with VKAs may be lower than with NOACs [4]. In clinical trials, reported proportions of premature OAC discontinuation within a period between two and three years ranged between 16.6% and 34.5% [5,6,7,8]. NOAC and VKA persistence were comparable before the end of the study in the Rivaroxaban Once Daily Oral Direct Factor Xa Inhibition Compared with Vitamin K Antagonism for Prevention of Stroke and Embolism Trial in Atrial Fibrillation (ROCKET AF) and Apixaban for the Prevention of Stroke in Subjects with Atrial Fibrillation (ARISTOTLE) trials [6,7]. The 1-year persistence of NOAC and VKA was 84.5% and 89.8%, respectively, in the Randomized Evaluation of Long-Term Anticoagulation Therapy (RE-LY) [5]. Among patients in clinical trials who permanently discontinued study drug, some of them started open-label OAC; hence, simply saying that these individuals “discontinue OAC” can be misleading.

Direct comparisons from prospectively collected data on OAC persistence in clinical practice are limited. The Global Registry on Long-Term Antithrombotic Treatment in Patients with Atrial Fibrillation (GLORIA-AF) is a large, ongoing global registry program, that enrolled patients with newly diagnosed non-valvular AF and >1 risk factor(s) for stroke. Based on phase III data from GLORIA-AF, which allows for comparisons of patients being prescribed NOACs vs. VKAs in clinical practice, we compared therapy persistence for NOACs and VKAs. In addition, we assessed the extent to which NOAC dosing (once daily (QD) vs. twice daily (BID)) affects drug persistence.

## 2. Materials and Methods

The design of GLORIA-AF (https://clinicaltrials.gov/ct2/home; trial registration numbers NCT01468701, NCT01671007, NCT01937377) has been previously published [9]. In brief, the GLORIA-AF study collects clinical practice evidence regarding patients with recently diagnosed AF to evaluate the safety and effectiveness of OACs. GLORIA-AF is a three-phase study. Phase I was performed before NOACs were available for stroke prevention in AF. Phase II started when dabigatran etexilate (dabigatran) was approved in participating countries. In Phase II, baseline characteristics of all enrolled patients were collected, and individuals who were treated with dabigatran were followed-up for 2 years. Phase III was conducted when dabigatran became more widely adopted. During phase III, follow-up data were collected for up to 3 years regardless of treatment, to examine the efficacy and safety of NOACs compared with VKAs [9]. This study enrolled consecutive patients with AF from 44 countries worldwide. Adult patients with non-valvular recently diagnosed AF (<3 months before the baseline visit) and at risk of stroke (CHA_2_DS_2_-VASc (congestive heart failure, hypertension, age ≥75 years, diabetes, stroke/transient ischaemic attack, vascular disease, age from 65 to 74 years, sex category) score ≥ 1) were included. Stroke and bleeding risks were evaluated using the CHA_2_DS_2_-VASc and HAS-BLED (hypertension, abnormal renal/liver function, stroke, bleeding history or predisposition, labile international normalized ratio, elderly (>65 years), drugs or alcohol concomitantly) scores [10,11]. Patients were managed according to routine standard of care and were not required to be prescribed any particular OAC or any OAC at all. This report is focused on 1-year follow-up of phase III patients, and includes data of all eligible patients who were anticoagulated.

Standard electronic case reports forms (eCRFs) were used to collect baseline characteristics and follow-up observation data. Initiation and discontinuation dates of OACs were recorded by the treating physician based on the patients’ source data records [3].

Baseline therapy was the treatment prescribed for long-term anticoagulation subsequent to the diagnosis of AF and recorded at the first visit. At follow-up visits (6, 12, 24, and 36 months after baseline), deviations from concomitant diseases, OAC changes, and all serious adverse events, interventions, and adverse drug reactions were collected.

### 2.1. Therapy Persistence

We defined persistence as remaining on therapy without a gap of longer than 30 days or a switch to an alternative therapy. Data on persistence were derived based on treatment information that was collected in eCRFs during the study. We measured the proportion of patients persisting on treatment after the first 12 months since beginning therapy.

### 2.2. Statistical Analysis

Categorical variables were summarized by frequencies and percentages, and continuous variables as mean and standard deviation (SD). Baseline characteristics included stratification of patients by type of OAC for stroke prevention (NOACs vs. VKAs) or daily treatment regimen (QD vs. BID). Descriptive analyses were performed for the subgroups of patients treated by any NOAC and VKA, as well as for patients on NOACs BID and QD. We used standardized differences to assess comparability [12]. Differences ≤10% were considered to reflect reasonable balance [13].

In the comparative analyses for 1-year treatment persistence, measured confounders were accounted by including these variables in the multivariable regression model. To reduce bias due to unmeasured confounders, we used asymmetrical propensity score (PS) trimming to exclude outliers from the study population before fitting the regression model. For example, for the NOAC vs. VKA comparison, per geographical region, we excluded patients with a PS below the 1.5th percentile of the PS distribution in the NOAC-exposed group and above the 98.5th percentile of the PS distribution in VKA-exposed group (i.e., patients from both groups with a propensity score less than the lower cut-off or greater than the higher cut-off were excluded) [14].

Multivariable Cox analyses were performed to adjust for differences in measured confounders (for all considered confounders, refer to Supplementary Tables S3 and S4). Missing data were handled using multiple imputation by the chained equations approach [15]. The imputation model was constructed using 54 baseline patient characteristics variables, including those used in the multivariable analyses (refer to Supplementary Table S5 for information on missing data). The number of imputations was 20. Each imputed dataset was analyzed separately, and results were averaged across the imputed datasets. Kaplan–Meier estimates of persistence were used to evaluate time to discontinuation as well as persistence rates at the end of each time period (6 and 12 months) based on trimmed patient sets. Similarly, all the preceding analyses were performed for the comparative analysis for 1-year persistence among patients on NOACs QD vs. BID. All analyses were performed using SAS software version 9.4 (SAS institute, Inc., Cary, NC, USA).

## 3. Results

Treatment models and baseline characteristics from GLORIA-AF have been previously reported [9]. In phase III (2014–2016), a total of 21,109 patients were eligible: 4808 (23%) patients were prescribed VKAs and took at least one dose, 12,577 (60%) patients received NOACs, 2344 (11%) patients were prescribed antiplatelet agents, 1372 (6%) patients received no antithrombotic treatment, and 8 (0%) individuals were prescribed other therapies. Of the 21,109 eligible patients, 4469 (21%) patients were prescribed apixaban, 3973 (19%) rivaroxaban, 3803 (18%) dabigatran, and 332 (1.6%) edoxaban. The majority of eligible patients were enrolled in Europe (51% in NOAC group and 57% in VKA group), followed by North America (28% vs. 15%), Asia (14% and 16%), and Latin America (7% and 12%).

### 3.1. Patient Characteristics

Patients’ mean age was 71.2 ± 10.3 years in the VKA group and 71.0 ± 10.2 years in the NOAC group. Approximately 56% of patients were male in each group (Supplementary Table S1). Baseline characteristics of patients who were on NOACs or VKAs are presented in Supplementary Table S1. The prevalence of paroxysmal AF in patients on VKAs and NOACs was 45% and 56%, respectively. In patients treated with VKAs, 41% suffered from persistent AF and 14% had permanent AF, as compared with 34% and 9.2%, respectively, in patients treated with NOACs. Congestive heart failure was more prevalent in the VKA group (29% vs. 20% in the NOAC group). Cardioversion was performed in 14% of individuals who were on VKAs and in 20% of individuals on NOACs (Supplementary Table S1).

Clinical demography, comorbid diseases, AF categorization, and concomitant treatments of patients on NOACs QD vs. NOACs BID are summarized in Supplementary Table S2. In patients on NOACs BID, the prevalence of previous stroke was 12% as compared with 7.6% in patients on NOACs QD. Cardioversion was performed in 23% of patients on NOACs QD and in 18% of patients on NOACs BID.

### 3.2. Overall Treatment Persistence (on Trimmed Patient Sets in Terms of Propensity Score)

**Figure 1:**
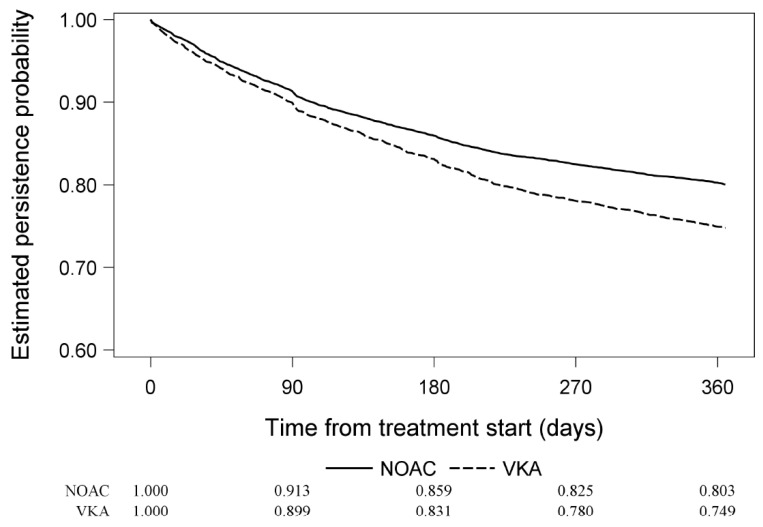
Drug persistence based on Kaplan–Meier estimator. Trimmed patients on VKAs and NOACs. NOACs, non-vitamin K antagonist oral anticoagulants; VKAs, vitamin K antagonists.

**Figure 2:**
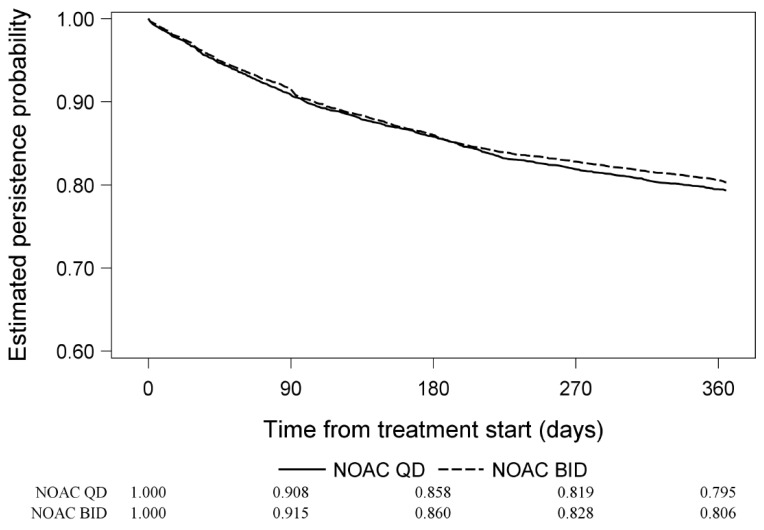
Drug persistence based on Kaplan–Meier estimator. Trimmed patients on NOACs once daily and NOACs twice daily. NOACs, non-vitamin K antagonist oral anticoagulants; VKAs, vitamin K antagonists.

The proportion of patients receiving NOACs who persisted on treatment was 85.9% at 6 months and 80.3% at 1 year. The corresponding proportions for patients receiving VKAs was 83.1% at 6 months and 74.9% at 1 year (Figure 1). Persistence of QD and BID NOAC treatment is shown in Figure 2.

At the end of the first year, 1090 (24%) patients had discontinued VKAs and 2303 (19%) patients had discontinued NOACs. In the same period, 822 (20%) patients had discontinued NOACs QD and 1488 (19%) patients had discontinued NOACs BID (Table 1).

### 3.3. Predictors of Treatment Persistence

From the multivariable analysis, persistence on NOACs was better than persistence on VKAs. We measured risk reduction using hazard ratios (HRs) for treatment discontinuation, with a HR < 1 indicating better persistence. We found a HR of 0.75 (95% CI 0.69–0.80) for NOACs (Table 2). Predictors of persistence included female gender (HR 0.92, 95% CI 0.86–1.00); a history of hypertension (HTN), (HR 0.91, 95% CI 0.84–0.99); age (65–75 years vs. <65 years), (HR 0.85, 95% CI 0.78–0.93); age (≥75 years vs. <65 years), (HR 0.81, 95% CI 0.72–0.90); alcohol use (<1 drink/week vs. never), (HR 0.90, 95% CI 0.83–0.99); type of AF (permanent vs. paroxysmal), (HR 0.83, 95% CI 0.73–0.95); categorization of AF (asymptomatic vs. symptomatic), (HR 0.73, 95% CI 0.67–0.80); categorization of AF (minimally symptomatic vs. symptomatic), (HR 0.82, 95% CI 0.76–0.89); body mass index (<18.5 kg/m^2^ vs. ≥18.5 kg/m^2^), (HR 0.94, 95% CI 0.88–1.00); and number of concomitant medications (≥median vs. <median), (HR 0.92, 95% CI 0.85–1.00) (Table 2).

Factors that predicted treatment discontinuation, for which the HR was >1, included Asian region (Asia vs. Europe), (HR 1.97, 95% CI 1.79–2.18); North American region (North America vs. Europe), (HR 1.53, 95% CI 1.40–1.68); use of medication predisposing to bleeding (antiplatelet therapy, inhibitors of cyclooxygenase 2, or other non-steroidal anti-inflammatory drugs (NSAIDs)), (HR 1.19, 95% CI 1.09–1.30); smoking status (current smoker vs. non-smoker), (HR 1.18, 95% CI 1.04–1.33); medical treatment reimbursement (private insurance vs. federal insurance), (HR 1.11, 95% CI 1.01–1.23); and use of proton pump inhibitors (PPIs), (HR 1.10, 95% CI 1.01–1.20).

A multivariable Cox regression model was used to analyze 1-year persistence with NOACs QD vs BID (Table 3). We measured risk reduction using HRs for treatment discontinuation, with a HR < 1 indicating better persistence. Therapy persistence did not differ much between NOACs QD and NOACs BID (HR 0.95, 95% CI 0.88–1.04). Predictors of persistence included age (65–74 years vs. <65 years), (HR 0.80, 95% CI 0.72–0.90); age (≥75 years vs. <65 years), (HR 0.80, 95% CI 0.70–0.91); female gender (HR 0.89, 95% CI 0.81–0.98); a history of HTN (HR 0.88, 95% CI 0.80–0.97); categorization of AF (minimally symptomatic vs. symptomatic), (HR 0.78, 95% CI 0.71–0.86); categorization of AF (asymptomatic vs. symptomatic), (HR 0.66, 95% CI, 0.60–0.74); and medical treatment reimbursement (self-pay/no coverage vs. federal insurance), (HR 0.67, 95% CI 0.54–0.84). Factors that predicted treatment discontinuation, for which the HR was >1, included use of antiplatelet therapy, inhibitors of cyclooxygenase 2, or other NSAIDs (HR 1.20, 95% CI 1.08–1.34); region (Asia vs. Europe), (HR 2.33, 95% CI 2.06–2.63); region (North America vs. Europe), (HR 1.71, 95% CI 1.53–1.91); and smoking status (current smoker vs. non-smoker), (HR 1.22, 95% CI 1.06–1.41).

## 4. Discussion

The main finding of this analysis of GLORIA-AF phase III dataset of patients with new onset non-valvular AF is that the 1-year probability of NOAC therapy persistence was better than with VKAs (80.3% and 74.9%, respectively). Second, for NOACs there was little difference between QD or BID regimes in terms of persistence (79.5% and 80.6%, respectively).

In our study, considering the first year after treatment initiation, NOAC persistence exceeded VKA persistence. Patients and physicians were aware of their participation in the clinical study, and this knowledge may have positively affected persistence via a Hawthorne effect [16]. However, OAC therapy persistence in GLORIA-AF study was closer to a “real-world” clinical practice than to randomized controlled trials. Despite lower cost of treatment with VKAs, regular monitoring of the VKA treatment, and possible deterioration of chronic kidney disease in some patients medicated with NOACs, VKA persistence remained lower than NOAC persistence.

The majority of previous studies addressing drug persistence consistently revealed higher persistence rates for NOACs than for VKAs at 6 months and 1 year [17,18,19,20]. Indeed, NOAC persistence at 1 year ranged from 38% to 99.7% and VKA persistence ranged from 24% to 93.2% [18,20,21,22,23,24].

We found persistence with NOACs to be nearly uniform regardless of the NOAC dosing regimen (QD vs BID): 85.8% and 86.0%, respectively, at 6 months. The proportion of patients persisting on NOAC therapy for 1 year was 79.5% using the QD regimen and 80.6% with the BID regimen. A number of other studies have directly examined persistence with particular NOACs and compared dosing regimens. In one study [25], 63.6% of patients were persistent with dabigatran therapy, while 68.1% of patients were persistent with rivaroxaban at 6 months; however, this study was retrospective and used administrative data from Canada, with a shorter follow-up period than that in our study. A retrospective analysis from the Danish National Patients Registry assessed persistence with OACs and found that after 1 year, 90% of rivaroxaban and 90% of apixaban patients were persistent, whereas 82% of dabigatran patients and 65% of warfarin patients were persistent [26]. In one Swedish study [27], the proportion of patients persisting on treatment based on incidence of prescription claims in intervals of six months was 86% with apixaban, 74% with dabigatran, and 77% with rivaroxaban at 1 year. In an Italian study based on administrative databases of five local healthcare units [28], the probability of drug persistence at 1 year since drug initiation was 75% among apixaban users, followed by 69% with rivaroxaban, 65% with dabigatran, and 43% with warfarin.

Many studies assessing persistence and adherence (defined as an active choice on the part of the patient to follow through with therapy) for NOACs revealed that the QD dosing regimen is associated with improved therapy persistence when compared with BID dosing regimen [29,30,31,32,33]. In our own study, treatment persistence was similar for QD and BID NOACs, maybe due to their similar tolerability and safety. Regardless of the studies, no NOAC guarantees ideal adherence and persistence. Hence, interventions found to improve persistence, including programs focusing on educating the patients, should be implemented [23]. This education is important given the clinical consequences of non-persistence or therapy changes [34].

In one systematic review [35], patients with asymptomatic chronic diseases were more compliant with QD than with BID dosing regimen. Another study [35] revealed that patients with HTN were also more compliant with QD than with the BID dosing regimen. Moreover, factors negatively associated with patient adherence (the extent to which the patient’s behavior matches the prescriber’s recommendations) to therapy and persistence were multiple daily dosing, chronic duration of disease, and asymptomatic disease. Non-valvular AF patients medicated with the QD dosing regimen for chronic medications had a higher probability of adherence and persistence compared with those on the BID dosing regimen [36].

Of importance, patients with AF treated with antiplatelet therapy, inhibitors of cyclooxygenase 2, other NSAIDs, or PPIs or being active smokers would have been regularly reminded by physicians about the significance of OACs in stroke prevention [3]. A lower persistence with OACs in patients with AF on PPIs could be related to therapy complications, such as bleeding. However, these results should not be over-interpreted given the short follow-up period of this study. In our study, occasional alcohol use was associated with better OAC persistence, but only for patients consuming <1 drink per week vs. never. Higher levels of alcohol consumption had no important association, implying that the alcohol association may be a chance finding.

The better treatment persistence in patients with AF and a history of HTN may be correlated with increased understanding of the significance of persistence with appropriately prescribed regimens [37,38]. Similar to our analysis, permanent AF and asymptomatic AF have been associated with improved persistence with dabigatran and they might be markers of disease severity [3]. In the prospective China-AF registry, factors such as asymptomatic AF were strong markers of worse warfarin persistence, while in the NOAC group, age < 75 years was associated with low persistence with NOACs [21]. However, patients consistently reminded of their disease state (i.e., by symptoms of AF) may be more likely to remain on therapy than those without such symptomatic reminders.

### 4.1. Strengths

In an observational setting, monitoring cannot be as comprehensive as in an interventional study setting. Nonetheless, in this study extensive measures were taken to ensure high data quality and complete reporting. These included on-site source data verification, regular review of aggregate data to mitigate quality concerns, and close and regular site contact to ensure comprehensive reporting.

### 4.2. Limitations

Our study had several important limitations. Patients consented to participate in the study and physicians were aware that persistence on treatment would be recorded. One of the study limitations is that registries are likely to enhance persistence by attracting patients with higher compliance. Frequent follow-up visits and the attention from the treating physician may influence and increase the OAC persistence. Studies evaluating persistence on anticoagulation should include a follow-up period of at least 1 year or longer, given the high risk of treatment discontinuation between 6 months and 1 year [39]. An even longer follow-up would help to assess more prolonged therapy persistence, for which information is limited.

## 5. Conclusions

The 1-year probability of drug persistence was better with NOACs than with VKAs. The NOAC dosing regimen (QD vs. BID) did not have important effect on 1-year therapy persistence.

## Figures and Tables

**Table 1:** Patients’ discontinuation of anticoagulants. Trimmed patient sets on VKAs, NOACs, NOACs once daily, and NOACs twice daily.

	VKAs			NOACs			NOACs QD			NOACs BID		
	<3 mo	3–6 mo	6–12 mo	<3 mo	3–6 mo	6–12 mo	<3 mo	3–6 mo	6–12 mo	<3 mo	3–6 mo	6–12 mo
Cumulative * number at risk, n	4478 (100.0)	3950 (88.2)	3602 (80.4)	11,945 (100.0)	10,748 (89.9)	9987 (83.6)	4097 (100.0)	3670 (89.6)	3422 (83.5)	7855 (100.0)	7076 (90.1)	6567 (83.6)
Cumulative * discontinued, n (%)	442 (9.9)	742 (16.6)	1090 (24.3)	1012 (8.5)	1649 (13.8)	2303 (19.3)	367 (9.0)	573 (14.0)	822 (20.1)	651 (8.3)	1079 (13.7)	1488 (18.9)
Cumulative * censored, n (%)	86 (1.9)	134 (3.0)	278 (6.2)	185 (1.6)	309 (2.6)	749 (6.3)	60 (1.5)	102 (2.5)	267 (6.5)	128 (1.6)	209 (2.7)	489 (6.2)

* The numbers are cumulative since the beginning of the first year. mo, months.

**Table 2:** Multivariate Cox regression model of 1-year persistence with NOACs or VKAs.

Variable	Hazard Ratio	95% Confidence Interval
NOACs vs. VKAs	0.75	0.69–0.80
Age (65–75 years vs. <65 years)	0.85	0.78–0.93
Age (≥75 years vs. <65 years)	0.81	0.72–0.90
Gender (female vs. male)	0.92	0.86–1.00
Hypertension (yes vs. no)	0.91	0.84–0.99
Type of atrial fibrillation (permanent vs. paroxysmal)	0.83	0.73–0.95
Medication usage predisposing to bleeding (yes vs. no)	1.19	1.09–1.30
Alcohol use (<1 drink/week vs. never)	0.90	0.83–0.99
Smoking status (current smoker vs. non-smoker)	1.18	1.04–1.33
Proton pump inhibitors (yes vs. no)	1.10	1.01–1.20
Categorization of atrial fibrillation (minimally symptomatic vs. symptomatic)	0.82	0.76–0.89
Categorization of atrial fibrillation (asymptomatic vs. symptomatic)	0.73	0.67–0.80
Region (Asia vs. Europe)	1.97	1.79–2.18
Region (North America vs. Europe)	1.53	1.40–1.68
Medical treatment reimbursement (private insurance vs. federal insurance)	1.11	1.01–1.23
Body mass index (<18.5 kg/m^2^ vs. ≥18.5 kg/m^2^)	0.94	0.88–1.00
Number of concomitant medications (≥median vs. <median)	0.92	0.85–1.00

The relevant descriptive data (including missing data for applicable variables) are presented in Supplementary Table S5. The remainder of covariates in the multivariable analysis is presented in Supplementary Table S3. NOACs, non-vitamin K antagonist oral anticoagulants, VKAs, vitamin K antagonists. The median number of concomitant medications is 3.

**Table 3:** Multivariate Cox regression model of 1-year persistence with NOACs once daily vs. twice daily.

Variable	Hazard Ratio	95% Confidence Interval
NOACs twice daily vs. once daily	0.95	0.88–1.04
Age (65–75 vs. <65)	0.80	0.72–0.90
Age (≥75 vs. <65)	0.80	0.70–0.91
Gender (female vs. male)	0.89	0.81–0.98
Hypertension (yes vs. no)	0.88	0.80–0.97
Medication usage predisposing to bleeding (yes vs. no)	1.20	1.08–1.34
Smoking status (current smoker vs. non-smoker)	1.22	1.06–1.41
Categorization of AF (minimally symptomatic vs. symptomatic)	0.78	0.71–0.86
Categorization of AF (asymptomatic vs. symptomatic)	0.66	0.60–0.74
Region (Asia vs. Europe)	2.33	2.06–2.63
Region (North America vs. Europe)	1.71	1.53–1.91
Medical treatment reimbursement (self-pay/no coverage vs. federal insurance)	0.67	0.54–0.84

AF, atrial fibrillation, NOACs, non-vitamin K antagonist oral anticoagulants. The relevant descriptive data (including missing data for applicable variables) are presented in Supplementary Table S6. The remainder of covariates in the multivariable analysis is presented in Supplementary Table S4. Medication usage predisposing to bleeding is defined as antiplatelet therapy, inhibitors of cyclooxygenase 2, or other non-steroidal anti-inflammatory drugs (NSAIDs).

## Supplementary Materials

The following are available online at https://www.mdpi.com/2077-0383/9/6/1969/s1, Table S1: Clinical characteristics of all eligible patients treated (or on) NOAC or VKA, Table S2: Baseline characteristics of all eligible patients on once daily regimen (rivaroxaban or edoxaban] or twice daily regimen (dabigatran or apixaban), Table S3: Multivariate Cox regression model of 1-year persistence with NOAC or VKA, Table S4: Multivariate Cox regression model of 1-year persistence with NOAC’s once daily vs. twice daily, Table S5: Clinical characteristics of restricted patients treated (or on) NOAC or VKA, Table S6: Baseline characteristics of restricted patients on once daily regimen (rivaroxaban or edoxaban] or twice daily regimen (dabigatran or apixaban).
