# Supplementary material for: Persistence with Anticoagulation for Atrial Fibrillation: Report from the GLORIA-AF Phase III 1-Year Follow-up
Source: J Clin Med. 2020 Jun 23;9(6):1969. doi: 10.3390/jcm9061969 (PMC7356563; doi:10.3390/jcm9061969)
Supplement: Supplementary file 1 [file jcm-09-01969-s001.pdf]

SUPPLEMENTARY MATERIAL

Persistence with Anticoagulation for Atrial fibrillation. Report from the GLORIA-AF Phase III 1-year follow-up

Monika Kozieł, MD, PhD, Michał Mazurek, MD, PhD, Christine Teutsch, MD, Hans-Christoph Diener, MD, PhD, Sergio J. Dubner, MD, Jonathan L. Halperin, MD, Chang-Sheng Ma, MD, Kenneth J. Rothman, DrPH, Axel Brandes, MD, Miney Paquette, MSc, Kristina Zint, MD, Lionel Riou França, PhD, Shihai Lu, PhD, Dorothee B. Bartels, PhD, Menno V. Huisman, MD, PhD, FESC, Gregory Y. H. Lip, MD

Table S1: Clinical characteristics of all eligible patients treated (or on) NOAC or VKA

	NOAC	VKA	Standardized difference
Patients, n (%)	**12,492 (100.0)**	**4,774 (100.0)**	
Age, mean (SD), yrs	**71.0 (10.2)**	**71.2 (10.3)**	**-0.014**
Age ≥ 75 yrs, n (%)	**4922 (39.4)**	**1928 (40.4)**	**-0.020**
Body mass index, mean (SD), kg/m2	**29.0 (6.6)**	**28.6 (6.1)**	**0.061**
Gender male, n (%)	**6849 (54.8)**	**2648 (55.5)**	**-0.013**
Type of atrial fibrillation, n (%)			
Paroxysmal	**7049 (56.4)**	**2149 (45.0)**	**0.230**
Persistent	**4290 (34.3)**	**1949 (40.8)**	**-0.134**
Permanent	**1153 (9.2)**	**676 (14.2)**	**-0.154**
Categorization of atrial fibrillation, n (%)			
Symptomatic	**3748 (30.0)**	**1602 (33.6)**	**-0.076**
Minimally symptomatic	**4139 (33.1)**	**1615 (33.8)**	**-0.015**
Asymptomatic	**4605 (36.9)**	**1557 (32.6)**	**0.089**
Medical history, n (%)			
Previous stroke	**1328 (10.6)**	**457 (9.6)**	**0.035**
Unknown	**6 (0.0)**	**1 (0.0)**	**0.015**
Myocardial infarction	**1048 (8.4)**	**542 (11.4)**	**-0.100**
Unknown	**9 (0.1)**	**3 (0.1)**	**0.004**

Coronary artery disease	**2110 (16.9)**	**910 (19.1)**	**-0.057**
Unknown	**280 (2.2)**	**164 (3.4)**	**-0.072**
Congestive heart failure	**2459 (19.7)**	**1365 (28.6)**	**-0.209**
Unknown	**94 (0.8)**	**45 (0.9)**	**-0.021**
Ejection fraction ≤40%	**1025 (8.2)**	**517 (10.8)**	**0.090**
Unknown	**290 (11.8)**	**204 (14.9)**	**-0.093**
Diabetes mellitus	**2905 (23.3)**	**1213 (25.4)**	**-0.050**
Unknown	**0 (0.0)**	**0 (0.0)**	
Prior bleeding	**609 (4.9)**	**248 (5.2)**	**-0.015**
Unknown	**282 (2.3)**	**65 (1.4)**	**0.067**
Renal impairment	**105 (0.8)**	**165 (3.5)**	**-0.181**
Unknown	**124 (1.0)**	**93 (1.9)**	**-0.079**
Hypertension, n (%)			
Uncontrolled hypertension	**1078 (8.6)**	**463 (9.7)**	**-0.037**
Controlled hypertension	**8267 (66.2)**	**3060 (64.1)**	**0.044**
Unknown	**170 (1.8)**	**79 (2.2)**	**-0.029**
Creatinine clearance (measured), mean (SD), ml/min	**83.0 (72.4)**	**76.9 (35.4)**	**0.109**
CHA_2DS_2-VASc score, median (Q1, Q3)	**3.0 (2.0, 4.0)**	**3.0 (2.0, 4.0)**	**-0.069**
HAS-BLED risk score, median (Q1, Q3)	**1.0 (1.0, 2.0)**	**1.0 (1.0, 2.0)**	**-0.014**
Cardioversion, n (%)	**2467 (19.7)**	**677 (14.2)**	**0.149**

Unknown	**134 (1.1)**	**38 (0.8)**	**0.029**
Atrial fibrillation ablation n, (%)	**248 (2.0)**	**81 (1.7)**	**0.022**
Unknown	**106 (0.8)**	**40 (0.8)**	**0.001**
Medications, n (%)			
Antiplatelet drug	**2172 (17.4)**	**897 (18.8)**	**-0.036**
Medication usage predisposing to bleeding	**2507 (20.1)**	**978 (20.5)**	**-0.010**
Number of concomitant medications, mean (SD)	**3.48 (1.80)**	**3.53 (1.84)**	**-0.027**
Physician specialty, n (%)			
Cardiologist	**10655 (85.3)**	**3907 (81.8)**	**0.093**
General practitioner / Primary care physician /Geriatrician	**580 (4.6)**	**218 (4.6)**	**0.004**
Internist	**407 (3.3)**	**316 (6.6)**	**-0.156**
Neurologist	**391 (3.1)**	**69 (1.4)**	**0.113**
Angiologist	**2 (0.0)**	**1 (0.0)**	**-0.004**
Other	**408 (3.3)**	**254 (5.3)**	**-0.102**
Missing	**49 (0.4)**	**9 (0.2)**	**0.038**
Medical treatment reimbursed by:			
Private insurance	**2034 (16.3)**	**482 (10.1)**	**0.184**
Statutory / federal insurance	**8972 (71.8)**	**3765 (78.9)**	**-0.164**
Self-pay / no coverage	**618 (4.9)**	**220 (4.6)**	**0.016**

Unknown	**868 (6.9)**	**307 (6.4)**	**0.021**
Region (N [%])			
Asia	**1794 (14.4)**	**787 (16.5)**	**-0.059**
Europe	**6354 (50.9)**	**2721 (57.0)**	**-0.123**
North America	**3483 (27.9)**	**725 (15.2)**	**0.313**
Latin America	**861 (6.9)**	**541 (11.3)**	**-0.155**
Race (N [%])			
Amer.Indian/Amer.Native/Alaska Native	**78 (0.6)**	**29 (0.6)**	**0.002**
Black/African American	**229 (1.8)**	**81 (1.7)**	**0.010**
White	**9205 (73.7)**	**3574 (74.9)**	**-0.027**
Asian	**1731 (13.9)**	**754 (15.8)**	**-0.055**
Native Hawaiian/Other Pacific Islander	**1 (0.0)**	**0 (0.0)**	
Arab/Middle East	**16 (0.1)**	**7 (0.1)**	**-0.005**
African	**6 (0.0)**	**2 (0.0)**	**0.002**
Missing	**847 (6.8)**	**195 (4.1)**	**0.119**
Smoking status (N [%])			
Non-smoker	**7065 (56.6)**	**2722 (57.0)**	**-0.009**
Current smoker	**1090 (8.7)**	**437 (9.2)**	**-0.015**
Past smoker	**3932 (31.5)**	**1477 (30.9)**	**0.012**
Unknown	**405 (3.2)**	**138 (2.9)**	**0.020**

Alcohol abuse (N [%])			
No (<8U/wk)	**10813 (86.6)**	**4176 (87.5)**	**-0.027**
Yes (>=8U/wk)	**937 (7.5)**	**303 (6.3)**	**0.046**
Unknown	**742 (5.9)**	**295 (6.2)**	**-0.010**
Alcohol use (N [%])			
Never	**5069 (40.6)**	**2149 (45.0)**	**-0.090**
<1 drink/week	**3078 (24.6)**	**1150 (24.1)**	**0.013**
1-7 drinks/week	**2666 (21.3)**	**877 (18.4)**	**0.075**
>=8 drinks/week	**937 (7.5)**	**303 (6.3)**	**0.046**
Unknown	**742 (5.9)**	**295 (6.2)**	**-0.010**
Type of site (N [%])			
General practice/primary care	**678 (5.4)**	**254 (5.3)**	**0.005**
Specialist office	**4060 (32.5)**	**1083 (22.7)**	**0.221**
Community hospital	**4111 (32.9)**	**1239 (26.0)**	**0.153**
University hospital	**3355 (26.9)**	**1922 (40.3)**	**-0.287**
Out-patient health care center	**98 (0.8)**	**162 (3.4)**	**-0.183**
Anticoagulation clinics	**45 (0.4)**	**57 (1.2)**	**-0.003**
Other	**145 (1.2)**	**57 (1.2)**	**-0.003**

Transient ischaemic attack (N [%])			
Yes	**634 (5.1)**	**196 (4.1)**	**0.046**
Unknown	**37 (0.3)**	**19 (0.4)**	**-0.017**
Pulmonary embolism (N [%])			
Yes	**84 (0.7)**	**25 (0.5)**	**0.019**
Unknown	**164 (1.3)**	**34 (0.7)**	**0.060**
Deep vein thrombosis (N [%])			
Yes	**157 (1.3)**	**65 (1.4)**	**-0.009**
Unknown	**190 (1.5)**	**38 (0.8)**	**0.068**
Angina pectoris (N [%])			
Yes	**971 (7.8)**	**440 (9.2)**	**-0.052**
Unknown	**122 (1.0)**	**48 (1.0)**	**-0.003**
Rheumatic Heart Disease (N [%])			
Yes	**45 (0.4)**	**31 (0.6)**	**-0.041**
Unknown	**154 (1.2)**	**31 (0.6)**	**0.061**
Additional antiplateletes (N [%])	**1691 (13.5)**	**726 (15.2)**	**-0.048**
Peripheral artery disease (N [%])			
Yes	**360 (2.9)**	**153 (3.2)**	**-0.019**
Unknown	**80 (0.6)**	**58 (1.2)**	**-0.060**
Cancer (N [%])			
Yes	**1320 (10.6)**	**471 (9.9)**	**0.023**

Unknown	**191 (1.5)**	**69 (1.4)**	**0.007**
Hyperlipidemia (N [%])			
Yes	**5233 (41.9)**	**1831 (38.4)**	**0.072**
Unknown	**286 (2.3)**	**141 (3.0)**	**-0.042**
Hepatic disease (N [%])			
Yes	**166 (1.3)**	**65 (1.4)**	**-0.003**
Unknown	**183 (1.5)**	**57 (1.2)**	**0.024**
Non-central nervous system arterial embolism (N [%])			
Yes	**48 (0.4)**	**21 (0.4)**	**-0.009**
Unknown	**42 (0.3)**	**11 (0.2)**	**0.019**
Presence of complex aortic plaque (N [%])			
Yes	**112 (0.9)**	**56 (1.2)**	**-0.027**
Unknown	**205 (1.6)**	**130 (2.7)**	**-0.074**

CHA_2DS_2-VASc, congestive heart failure, hypertension, age ≥ 75 years, diabetes, stroke/ transient ischaemic attack, vascular disease, age 65 to 74 years, sex category, HAS-BLED, hypertension, abnormal renal/ liver function, stroke, bleeding history or predisposition, labile International Normalised Ratio, elderly, drugs or alcohol concomitantly, NOAC, non-vitamin K antagonist oral anticoagulants, SD, standard deviation, VKA, vitamin K antagonists. Medication usage predisposing to bleeding is defined as antiplatelet therapy or inhibitors of cyclooxygenase 2 or other non-steroidal anti-inflammatory drugs (NSAIDs).

Table S2: **Baseline characteristics of all eligible patients on once daily regimen (rivaroxaban or edoxaban) or twice daily regimen (dabigatran or apixaban)**

	**Once daily**	**Twice daily**	**Standardized difference**
Patients, n (%)	**4277 (100.0)**	**8215 (100.0)**	
Age, mean (SD), yrs	**70.5 (10.2)**	**71.3 (10.2)**	**-0.077**
Age ≥ 75 yrs, n (%)	**1605 (37.5)**	**3317 (40.4)**	**-0.059**
Body mass index, mean (SD), kg/m2	**29.4 (6.5)**	**28. 8 (6. 6)**	**0.082**
Gender, male, n (%)	**2399 (56.1)**	**4450 (54.2)**	**0.039**
Type of atrial fibrillation, n (%)			
Paroxysmal	**2439 (57.0)**	**4610 (56.1)**	**0.018**
Persistent	**1486 (34.7)**	**2804 (34.1)**	**0.013**
Permanent	**352 (8.2)**	**801 (9.8)**	**-0.053**
Categorization of atrial fibrillation, n (%)			
Symptomatic	**1312 (30.7)**	**2436 (29.7)**	**0.022**
Minimally symptomatic	**1410 (33.0)**	**2729 (33.2)**	**-0.005**
Asymptomatic	**1555 (36.4)**	**3050 (37.1)**	**-0.016**
Medical history, n (%)			
Previous stroke	**326 (7.6)**	**1002 (12.2)**	**-0.154**
Unknown	**2 (0.0)**	**4 (0.0)**	**-0.001**
Myocardial infarction	**366 (8.6)**	**682 (8.3)**	**0.009**
Unknown	**3 (0.1)**	**6 (0.1)**	**-0.001**

Coronary artery disease	**745 (17.4)**	**1365 (16.6)**	**0.021**
Unknown	**132 (3.1)**	**148 (1.8)**	**0.083**
Congestive heart failure	**846 (19.8)**	**1613 (19.6)**	**-0.004**
Unknown	**37 (0.9)**	**57 (0.7)**	**0.019**
Ejection fraction ≤ 40%	**351 (8.2)**	**674 (8.2)**	**0.000**
Unknown	**85 (10.0)**	**205 (12.7)**	**-0.084**
Diabetes mellitus	**1052 (24.6)**	**1853 (22.6)**	**0.048**
Unknown	**0 (0.0)**	**0 (0.0)**	
Prior bleeding	**219 (5.1)**	**390 (4.7)**	**0.017**
Unknown	**124 (2.9)**	**158 (1.9)**	**0.064**
Renal impairment	**29 (0.7)**	**76 (0.9)**	**-0.028**
Unknown	**56 (1.3)**	**68 (0.8)**	**0.047**
Hypertension, n (%)			
Uncontrolled hypertension	**311 (7.3)**	**767 (9.3)**	**-0.075**
Controlled hypertension	**2872 (67.1)**	**5395 (65.7)**	**-0.031**
Unknown	**53 (1.6)**	**117 (1.9)**	**-0.017**
Creatinine clearance (measured), mean (SD), ml/min	**85.0 (40.1)**	**82.0 (84.8)**	**0.046**
CHA_2DS_2-VASc score, median (Q1, Q3)	**3.0 (2.0, 4.0)**	**3.0 (2.0, 4.0)**	**-0.116**
HAS-BLED risk score, median (Q1, Q3)	**1.0 (1.0, 2.0)**	**1.0 (1.0, 2.0)**	**-0.075**
Cardioversion, n (%)	**967(22.6)**	**1500 (18.3)**	**0.108**

Unknown	**43 (1.0)**	**91 (1.1)**	**-0.010**
Atrial fibrillation ablation, n (%)	**59 (1.4)**	**189 (2.3)**	**-0.069**
Unknown	**34 (0.8)**	**72 (0.9)**	**-0.009**
Medications, n (%)			
Antiplatelet drug	**777 (18.2)**	**1395 (17.0)**	**0.312**
Medication usage predisposing to bleeding	**906 (21.2)**	**1601 (19.5)**	**0.042**
Number of concomitant medications, mean (SD)	**3.52 (1.84)**	**3.47 (1.78)**	**0.029**
Physician specialty, n (%)			
Cardiologist	**3709 (86.7)**	**6946 (84.6)**	**0.062**
General practitioner / Primary care physician /Geriatrician	**203 (4.7)**	**377 (4.6)**	**0.007**
Internist	**149 (3.5)**	**258 (3.1)**	**0.019**
Neurologist	**64 (1.5)**	**327 (4.0)**	**-0.153**
Angiologist	**1 (0.0)**	**1 (0.0)**	**0.008**
Other	**142 (3.3)**	**266 (3.2)**	**0.005**
Missing	**9 (0.2)**	**40 (0.5)**	**-0.047**
Region (N [%])			
Asia	**503 (11.8)**	**1291 (15.7)**	**-0.115**
Europe	**2156 (50.4)**	**4198 (51.1)**	**-0.014**
North America	**1336 (31.2)**	**2147 (26.1)**	**0.113**
Latin America	**282 (6.6)**	**579 (7.0)**	**-0.018**

Race (N [%])			
Amer.Indian/Amer.Native/Alaska Native	**31 (0.7)**	**47 (0.6)**	**0.019**
Black/African American	**69 (1.6)**	**160 (1.9)**	**-0.025**
White	**3277 (76.6)**	**5928 (72.2)**	**0.102**
Asian	**501 (11.7)**	**1230 (15.0)**	**-0.096**
Native Hawaiian/Other Pacific Islander	**0 (0.0)**	**1 (0.0)**	
Arab/Middle East	**7 (0.2)**	**9 (0.1)**	**-0.015**
African	**2 (0.0)**	**4 (0.0)**	**-0.001**
Missing	**254 (5.9)**	**593 (7.2)**	**-0.052**
Smoking status (N [%])			
Non-smoker	**2349 (54.9)**	**4716 (57.4)**	**-0.050**
Current smoker	**343 (8.0)**	**747 (9.1)**	**-0.038**
Past smoker	**1440 (33.7)**	**2492 (30.3)**	**0.071**
Unknown	**145 (3.4)**	**260 (3.2)**	**0.013**
Alcohol abuse (N [%])			
No (<8U/wk)	**3674 (85.9)**	**7139 (86.9)**	**-0.029**
Yes (>=8U/wk)	**345 (8.1)**	**592 (7.2)**	**0.032**
Unknown	**258 (6.0)**	**484 (5.9)**	**0.006**
Alcohol use (N [%])			
Never	**1620 (37.9)**	**3449 (42.0)**	**-0.084**
<1 drink/week	**1101 (25.7)**	**1977 (24.1)**	**0.039**
1-7 drinks/week	**953 (22.3)**	**1713 (20.9)**	**0.035**

>=8 drinks/week	**345 (8.1)**	**592 (7.2)**	**0.032**
Unknown	**258 (6.0)**	**484 (5.9)**	**0.006**
Type of site (N [%])			
General practice /primary care	**256 (6.0)**	**422 (5.1)**	**0.037**
Specialist office	**1536 (35.9)**	**2524 (30.7)**	**0.110**
Community hospital	**1337 (31.3)**	**2774 (33.8)**	**-0.054**
University hospital	**1034 (24.2)**	**2321 (28.3)**	**-0.093**
Out-patient health care center	**28 (0.7)**	**70 (0.9)**	**-0.023**
Anticoagulation clinics	**19 (0.4)**	**26 (0.3)**	**0.021**
Transient ischaemic attack (N [%])			
Yes	**189 (4.4)**	**445 (5.4)**	**-0.046**
Unknown	**12 (0.3)**	**25 (0.3)**	**-0.004**
Pulmonary embolism (N [%])			
Yes	**40 (0.9)**	**44 (0.5)**	**0.047**
Unknown	**71 (1.7)**	**93 (1.1)**	**0.045**
Deep vein thrombosis (N [%])			
Yes	**62 (1.4)**	**95 (1.2)**	**0.026**
Unknown	**81 (1.9)**	**109 (1.3)**	**0.045**
Angina pectoris (N [%])			
Yes	**298 (7.0)**	**673 (8.2)**	**-0.046**
Unknown	**61 (1.4)**	**61 (0.7)**	**0.066**
Rheumatic Heart Disease (N [%])			

Yes	**11 (0.3)**	**34 (0.4)**	**-0.027**
Unknown	**81 (1.9)**	**73 (0.9)**	**0.086**
Additional antiplatelets (N [%])			
	**608 (14.2)**	**1083 (13.2)**	**0.300**
Peripheral artery disease (N [%])			
Yes	**118 (2.8)**	**242 (2.9)**	**-0.011**
Unknown	**30 (0.7)**	**50 (0.6)**	**0.012**
Cancer (N [%])			
Yes	**441 (10.3)**	**879 (10.7)**	**-0.013**
Unknown	**90 (2.1)**	**101 (1.2)**	**0.068**
Hyperlipidemia (N [%])			
Yes	**1815 (42.4)**	**3418 (41.6)**	**0.017**
Unknown	**116 (2.7)**	**170 (2.1)**	**0.042**
Hepatic disease (N [%])			
Yes	**65 (1.5)**	**101 (1.2)**	**0.025**
Unknown	**78 (1.8)**	**105 (1.3)**	**0.044**
Non-central nervous system arterial embolism (N [%])			
Yes	**17 (0.4)**	**31 (0.4)**	**0.003**
Presence of complex aortic plaque (N [%])			
Yes	**32 (0.7)**	**80 (1.0)**	**-0.024**
Proton Pump inhibitors (N [%])			

Yes	**1073 (25.1)**	**2113 (25.7)**	**-0.015**
Chronic gastrointestinal diseases (N [%])			
Yes	**639 (14.9)**	**1094 (13.3)**	**0.047**
Unknown	**79 (1.8)**	**117 (1.4)**	**0.033**
Vascular disease (N [%])			
Yes	**479 (11.2)**	**938 (11.4)**	**-0.007**
Dementia (N [%])			
Yes	**25 (0.6)**	**54 (0.7)**	**-.009**
Missing	**81 (1.9)**	**96 (1.2)**	**0.059**

CHA_2DS_2-VASc, congestive heart failure, hypertension, age ≥ 75 years, diabetes, stroke/ transient ischaemic attack, vascular disease, age 65 to 74 years, sex category, HAS-BLED, hypertension, abnormal renal/ liver function, stroke, bleeding history or predisposition, labile International Normalised Ratio, elderly, drugs or alcohol concomitantly, NOAC, non-vitamin K antagonist oral anticoagulants, SD, standard deviation, VKA, vitamin K antagonists.

Medication usage predisposing to bleeding is defined as antiplatelet therapy or inhibitors of cyclooxygenase 2 or other non-steroidal anti-inflammatory drugs (NSAIDs).

Table S3: Multivariate Cox regression model of 1-year persistence with NOAC or VKA

		95% CI	
**Parameter**	**Hazard ratio**	**Lower limit**	**Upper limit**
Myocardial infarction (MI) (yes vs. no)	0.81	0.65	1.01
Congestive heart failure (yes vs. no)	0.93	0.85	1.01
Diabetes mellitus (Inclusion criteria) (yes vs. no)	0.95	0.88	1.04
Previous stroke (yes vs. no)	0.96	0.86	1.08
Creatinine clearance (measured) class 1 (<30 vs. >=80)	1.14	0.84	1.53
Creatinine clearance (measured) class 1 (30-<50 vs. >=80)	1.03	0.90	1.19
Creatinine clearance (measured) class 1 (50-<80 vs. >=80)	0.99	0.90	1.08
Type of AF (Persistent vs. paroxysmal)	1.00	0.93	1.08
Prior bleeding (yes vs. no)	1.14	0.98	1.33
Hepatic disease (yes vs. no)	1.08	0.81	1.43
Alcohol use (1-7 drinks/week vs. never)	0.97	0.88	1.07
Alcohol use (>=8 drinks/week vs. never)	0.91	0.79	1.05
Smoking status (Past smoker vs. non-smoker)	1.08	0.99	1.17
Coronary artery disease (CAD) (yes vs. no)	1.01	0.91	1.13
Chronic gastrointestinal diseases (yes vs. no)	0.99	0.89	1.09
Cancer (yes vs. no)	1.08	0.97	1.21

		95% CI	
**Parameter**	**Hazard ratio**	**Lower limit**	**Upper limit**
Dementia (yes vs. no)	1.07	0.66	1.72
Vascular disease defined as prior myocardial infarction, peripheral artery disease, complex aortic plaque (yes vs. no)	1.17	0.97	1.41
Systolic blood pressure (>160 mmHg vs. <=160 mmHg)	0.96	0.82	1.11

AF; atrial fibrillation

Table S4: Multivariate Cox regression model of 1-year persistence with NOAC's once daily vs twice daily

		95% CI	
Parameter	Hazard ratio	Lower limit	Upper limit
Diabetes mellitus (inclusion criteria) (yes vs. no)	0.94	0.85	1.05
Prior bleeding (yes vs. no)	1.18	0.98	1.42
Previous stroke (yes vs. no)	0.93	0.8	1.08
Congestive heart failure (yes vs. no)	0.97	0.87	1.08
Creatinine clearance (measured) class 1 (<30 vs. >=80)	1.06	0.71	1.59
Creatinine clearance (measured) class 1 (30-<50 vs. >=80)	1.01	0.85	1.20
Creatinine clearance (measured) class 1 (50-<80 vs. >=80)	0.93	0.83	1.04
Type of AF (persistent vs. paroxysmal)	1.00	0.92	1.10
Type of AF (permanent vs. paroxysmal)	0.92	0.78	1.09
Hepatic disease (yes vs. no)	1.05	0.76	1.47
Alcohol use (<1 drink / week vs. never)	0.91	0.81	1.01
Alcohol use (1-7 drinks/week vs. never)	0.94	0.84	1.05
Alcohol use (>=8 drinks/week vs. never)	0.90	0.76	1.07
Smoking status (Past smoker vs. non-smoker)	1.04	0.94	1.15
Proton Pump inhibitors (yes vs. no)	1.10	0.99	1.22

		95% CI	
Parameter	Hazard ratio	Lower limit	Upper limit
Coronary artery disease (CAD) (yes vs. no)	1.11	0.98	1.27
Chronic gastrointestinal diseases (yes vs. no)	0.91	0.81	1.03
Medical treatment reimbursed by (private insurance vs. statutory/federal insurance)	1.06	0.95	1.20
Body mass index (<18.5 kg/m^2 vs ≥ 18.5 kg/m^2)	0.94	0.87	1.02
Number of concomitant medications (>= median vs. < median)	0.93	0.84	1.03
Cancer (yes vs. no)	1.06	0.93	1.21
Dementia (yes vs. no)	0.90	0.51	1.58
Vascular disease defined as prior myocardial infarction, peripheral artery disease, complex aortic plaque (yes vs. no)	1.11	0.87	1.43
Systolic blood pressure (>160 mmHg vs. <=160 mmHg)	1.10	0.92	1.31

AF; atrial fibrillation

Table S5: Clinical characteristics of restricted patients treated (or on) NOAC or VKA

	NOAC	VKA	Standardized difference
Patients, n (%)	**11945**	**4478**	
Age, mean (SD), yrs	**71.1 (10.2)**	**71.2 (10.3)**	**-0.013**
Age ≥ 75 yrs, n (%)	**4711 (39.4)**	**1819 (40.6)**	**-0.024**
Body mass index, mean (SD), kg/m2	**29.0 (6.6)**	**28.6 (6.1)**	**0.064**
Gender male, n (%)	**6473 (54.2)**	**2446 (54.6)**	**-0.009**
Type of atrial fibrillation, n (%)			
Paroxysmal	**6736 (56.4)**	**2002 (44.7)**	**0.235**
Persistent	**4091 (34.2)**	**1831 (40.9)**	**-0.137**
Permanent	**1118 (9.4)**	**645 (14.4)**	**-0.156**
Categorization of atrial fibrillation, n (%)			
Symptomatic	**3550 (29.7)**	**1484 (33.1)**	**-0.074**
Minimally symptomatic	**3986 (33.4)**	**1540 (34.4)**	**-0.022**
Asymptomatic	**4409 (36.9)**	**1454 (32.5)**	**0.093**
Medical history, n (%)			
Previous stroke	**1252 (10.5)**	**412 (9.2)**	**0.043**
Unknown	**6 (0.1)**	**1 (0.0)**	**0.015**
Myocardial infarction	**972 (8.1)**	**464 (10.4)**	**-0.77**
Unknown	**8 (0.1)**	**1 (0.0)**	**0.021**

Coronary artery disease	**1963 (16.4)**	**792 (17.7)**	**-0.033**
Unknown	**253 (2.1)**	**150 (3.3)**	**-0.076**
Congestive heart failure	**2308 (19.3)**	**1248 (27.9)**	**-0.202**
Unknown	**86 (0.7)**	**27 (0.8)**	**-0.12**
Ejection fraction ≤40%	**964 (41.8)**	**463 (37.1)**	**0.096**
Unknown	**274 (11.9)**	**190 (15.2)**	**-0.098**
Diabetes mellitus	**2762 (23.1)**	**1109 (24.8)**	**-0.039**
Unknown	**0 (0.0)**	**0 (0.0)**	
Prior bleeding	**572 (4.8)**	**220 (4.9)**	**-0.006**
Unknown	**255 (2.1)**	**52 (1.2)**	**0.077**
Renal impairment	**19 (0.2)**	**18 (0.4)**	**-0.046**
Unknown	**110 (0.9)**	**81 (1.8)**	**-0.077**
Hypertension, n (%)			
Uncontrolled hypertension	**930 (10.3)**	**385 (11.5)**	**-0.039**
Controlled hypertension	**7984 (88.3)**	**2904 (86.7)**	**0.048**
Unknown	**0 (0.0)**	**0 (0.0))**	
Creatinine clearance (measured), mean (SD), ml/min	**83.3 (73.6)**	**78.2 (34.5)**	**0.089**
CHA_2DS_2-VASc score, median (Q1, Q3)	**3.0 (2.0, 4.0)**	**3.0 (2.0, 4.0)**	**-0.057**
HAS-BLED risk score, median (Q1, Q3)	**1.0 (1.0, 2.0)**	**1.0 (1.0, 2.0)**	**-0.018**
Cardioversion, n (%)	**2354 (19.7)**	**627 (14.0)**	**0.153**

Unknown	**122 (1.0)**	**33 (0.7)**	**0.031**
Atrial fibrillation ablation n, (%)	**234 (2.0)**	**69 (1.5)**	**0.032**
Unknown	**96 (0.8)**	**35 (0.8)**	**0.002**
Medications, n (%)			
Antiplatelet drug	**2064 (17.3)**	**812 (18.1)**	**-0.022**
Medication usage predisposing to bleeding	**2383 (19.9)**	**888 (19.8)**	**0.003**
Number of concomitant medications, mean (SD)	**3.5 (1.8)**	**3.5 (1.8)**	**-0.015**
Physician specialty, n (%)			
Cardiologist	**10175 (85.2)**	**3677 (82.1)**	**0.083**
General practitioner / Primary care physician /Geriatrician	**559 (4.7)**	**199 (4.4)**	**0.011**
Internist	**397 (3.3)**	**297 (6.6)**	**-0.153**
Neurologist	**375 (3.1)**	**60 (1.3)**	**0.122**
Angiologist	**2 (0.0)**	**1 (0.0)**	**-0.004**
Other	**389 (3.3)**	**236 (5.3)**	**-0.099**
Missing	**48 (0.4)**	**8 (0.2)**	**0.042**
Medical treatment reimbursed by:			
Private insurance	**1919 (16.1)**	**439 (9.8)**	**0.187**
Statutory / federal insurance	**8619 (72.2)**	**3546 (79.2)**	**-0.164**
Self-pay / no coverage	**819 (6.9)**	**289 (6.5)**	**0.016**
Unknown	**819 (6.9)**	**289 (6.5)**	**0.016**

Region (N [%])			
Asia	**1644 (13.8)**	**730 (16.3)**	**-0.071**
Europe	**6149 (51.5)**	**2575 (57.5)**	**-0.121**
North America	**3345 (28.0)**	**666 (14.9)**	**0.324**
Latin America	**807 (6.8)**	**507 (11.3)**	**-0.159**
Race (N [%])			
Amer.Indian/Amer.Native/Alaska Native	**76 (0.6)**	**27 (0.6)**	**0.004**
Black/African American	**222 (1.9)**	**70 (1.6)**	**0.023**
White	**8862 (74.2)**	**3379 (75.5)**	**-0.029**
Asian	**1587 (13.3)**	**698 (15.6)**	**-0.066**
Native Hawaiian/Other Pacific Islander	**1 (0.0)**	**0 (0.0)**	
Arab/Middle East	**16 (0.1)**	**6 (0.1)**	**0.000**
African	**5 (0.0)**	**1 (0.0)**	**0.011**
Missing	**824 (6.9)**	**176 (3.9)**	**0.131**
Smoking status (N [%])			
Non-smoker	**6794 (56.9)**	**2576 (57.5)**	**-0.013**
Current smoker	**1028 (8.6)**	**405 (9.0)**	**-0.015**
Past smoker	**3765 (31.5)**	**1371 (30.6)**	**0.019**
Unknown	**358 (3.0)**	**126 (2.8)**	**0.011**
Alcohol abuse (N [%])			
No (<8U/wk)	**10441 (87.4)**	**3932 (87.8)**	**-0.012**

Yes (>=8U/wk)	**835 (7.0)**	**279 (6.2)**	**0.031**
Unknown	**669 (5.6)**	**267 (6.0)**	**-0.016**
Alcohol use (N [%])			
Never	**4885 (40.9)**	**2014 (45.0)**	**-0.083**
<1 drink/week	**2978 (24.9)**	**1088 (24.3)**	**0.015**
1-7 drinks/week	**2578 (21.6)**	**830 (18.5)**	**0.076**
>=8 drinks/week	**835 (7.0)**	**279 (6.2)**	**0.031**
Unknown	**669 (5.6)**	**267 (6.0)**	**-0.016**
Type of site (N [%])			
General practice/primary care	**648 (5.4)**	**233 (5.2)**	**0.009**
Specialist office	**3912 (32.8)**	**1012 (22.6)**	**0.228**
Community hospital	**3939 (33.0)**	**1162 (25.9)**	**0.155**
University hospital	**3166 (26.5)**	**1809 (40.4)**	**-0.298**
Out-patient health care center	**98 (0.8)**	**155 (3.5)**	**-0.183**
Anticoagulation clinics	**41 (0.3)**	**51 (1.1)**	**-0.093**

Transient ischaemic attack (N [%])	**606 (5.1)**	**185 (4.1)**	**0.045**
Unknown	**6 (0.1)**	**1 (0.0)**	**0.014**
Pulmonary embolism (N [%])	**81 (0.7)**	**24 (0.5)**	**0.018**
Unknown	**139 (1.2)**	**27 (0.6)**	**0.060**
Deep vein thrombosis (N [%])	**143 (1.2)**	**62 (1.4)**	**-0.017**
Unknown	**253 (2.1)**	**150 (3.3)**	**0.065**
Angina pectoris (N [%])	**897 (7.5)**	**391 (8.7)**	**-0.045**
Unknown	**102 (0.9)**	**41 (0.9)**	**-0.006**
Rheumatic heart disease (N [%])	**44 (0.4)**	**29 (0.6)**	**-0.39**
Unknown	**126 (1.1)**	**21 (0.5)**	**0.067**
Additional antiplatelets (N [%])	**1602 (13.4)**	**563 (14.6)**	**-0.034**
Peripheral artery disease (N [%])	328 (2.7)	125 (2.8)	-0.003
Unknown	**76 (0.6)**	**47 (1.0)**	**-0.045**
Cancer (N [%])	**1259 (10.5)**	**435 (9.7)**	**0.027**
Unknown	**170 (1.4)**	**59 (1.3)**	**0.009**
Hyperlipidemia (N [%])	4986 (41.7)	1698 (37.9)	0.008
Unknown	**265 (2.2)**	**129 (2.9)**	**-0.042**
Hepatic disease (N [%])	**117 (1.0)**	**50 (1.1)**	**-0.014**
Unknown	**163 (1.4)**	**49 (1.1)**	**0.025**

Non-central nervous system arterial embolism (N [%])	**46 (0.4)**	**21 (0.5)**	**-0.013**
Unknown	**36 (0.3)**	**9 (0.2)**	**0.020**
Presence of complex aortic plaque (N [%])	**101 (0.8)**	**44 (1.0)**	**-0.014**
Unknown	**189 (1.6)**	**103 (2.3)**	**-0.052**

CHA_2DS_2-VASc, congestive heart failure, hypertension, age ≥ 75 years, diabetes, stroke/transient ischaemic attack, vascular disease, age 65 to 74 years, sex category, HAS-BLED, hypertension, abnormal renal/liver function, stroke, bleeding history or predisposition, labile International Normalised Ratio, elderly, drugs or alcohol concomitantly, NOAC, non-vitamin K antagonist oral anticoagulants, SD, standard deviation, VKA, vitamin K antagonists.

Medication usage predisposing to bleeding is defined as antiplatelet therapy or inhibitors of cyclooxygenase 2 or other non-steroidal anti-inflammatory drugs (NSAIDs).

Table S6: **Baseline characteristics of restricted patients on once daily regimen (rivaroxaban or edoxaban) or twice daily regimen (dabigatran or apixaban)**

	**Once daily**	**Twice daily**	**Standardized difference**
Patients, n (%)	**4097**	**7855**	
Age, mean (SD), yrs	70.6 (10.2)	71.3 (10.1)	-0.073
Age ≥ 75 yrs, n (%)	1545 (37.7)	3164 (40.3)	-0.053
Body mass index, mean (SD), kg/m2	29.4 (6.5)	28.8 (6.6)	0.088
Gender, male, n (%)	2268 (55.4)	4209 (53.6)	0.036
Type of atrial fibrillation, n (%)			
Paroxysmal	2339 (57.1)	4425 (56.3)	0.015
Persistent	1415 (34.5)	2660 (33.9)	0.014
Permanent	343 (8.4)	770 (9.8)	-0.050
Categorization of atrial fibrillation, n (%)			
Symptomatic	1253 (30.6)	2315 (29.5)	0.024
Minimally symptomatic	1353 (33.0)	2618 (33.3)	-0.007
Asymptomatic	1491 (36.4)	2922 (37.2)	-0.017
Medical history, n (%)			
Previous stroke	305 (7.4)	950 (12.1)	-0.157
Unknown	2 (0.0)	4 (0.1)	-0.001
Myocardial infarction	336 (8.2)	631 (8.0)	0.006
Unknown	2 (0.0)	4 (0.1)	-0.001

Coronary artery disease	696 (17.0)	1283 (16.3)	0.018
Unknown	119 (2.9)	131 (1.7)	0.083
Congestive heart failure	783 (19.1)	1519 (19.3)	-0.006
Ejection fraction ≤ 40%	322 (41.1)	632 (41.6)	0.010
Unknown	79 (10.1)	193 (12.7)	-0.082
Diabetes mellitus	968 (23.6)	1729 (22.0)	0.039
Unknown	0 (0.0)	0 (0.0)	
Prior bleeding	203 (5.0)	360 (4.6)	0.017
Unknown	110 (2.7)	144 (1.8)	0.057
Renal impairment	14 (0.3)	19 (0.2)	0.019
Unknown	48 (1.2)	64 (0.8)	0.036
Hypertension, n (%)			
Uncontrolled hypertension	281 (6.9)	646 (8.2)	-0.052
Controlled hypertension	2766 (67.5)	5232 (66.6)	0.019
Unknown	51 (1.6)	109 (1.8)	-0.013
Creatinine clearance (measured), mean (SD), ml/min	84.8 (39.4)	82.1 (36.2)	0.040
CHA_2DS_2-VASc score, median (Q1, Q3)	3.0 (2.0, 4.0)	3.0 (2.0, 4.0)	-0.117
HAS-BLED risk score, median (Q1, Q3)	1.0 (1.0, 2.0)	1.0 (1.0, 2.0)	-0.063
Cardioversion, n (%)	926 (22.6)	1427 (18.2)	0.110
Unknown	40 (0.1)	86 (1.1)	-0.012

Atrial fibrillation ablation, n (%)	57 (1.4)	170 (2.2)	-0.059
Unknown	32 (0.8)	68 (0.9)	-0.009
Medications, n (%)			
Antiplatelet drug	738 (18.0)	1303 (16.6)	0.038
Medication usage predisposing to bleeding	859 (21.0)	1495 (19.0)	0.048
Number of concomitant medications, mean (SD)	3.50 (1.8)	3.45 (1.8)	0.029
Physician specialty, n (%)			
Cardiologist	3550 (86.6)	6647 (84.6)	0.058
General practitioner / Primary care physician /Geriatrician	197 (4.8)	356 (4.5)	0.013
Internist	144 (3.5)	250 (3.2)	0.018
Neurologist	61 (1.5)	312 (4.0)	-0.153
Angiologist	1 (0.0)	1 (0.0)	0.009
Other	136 (3.3)	250 (3.2)	0.008
Missing	8 (0.2)	39 (0.5)	-0.051
Region (N [%])			
Asia	470 (11.5)	1228 (15.6)	-0.122
Europe	2064 (50.4)	4039 (51.4)	-0.021
North America	1294 (31.6)	2044 (26.0)	0.123
Latin America	269 (6.6)	544 (6.9)	-0.014
Race (N [%])			

Amer.Indian/Amer.Native/Alaska Native	31 (0.8)	47 (0.6)	0.019
Black/African American	66 (1.6)	148 (1.9)	-0.021
White	3153 (77.0)	5671 (72.2)	0.109
Asian	468 (11.4)	1175 (15.0)	-0.105
Native Hawaiian/Other Pacific Islander	0 (0.0)	1 (0.0)	
Arab/Middle East	7 (0.2)	9 (0.1)	0.015
African	2 (0.0)	4 (0.1)	-0.001
Smoking status (N [%])			
Non-smoker	2277 (55.6)	4548 (57.9)	-0.047
Current smoker	324 (7.9)	699 (8.9)	-0.036
Past smoker	1357 (33.1)	2357 (30.0)	0.067
Unknown	139 (3.4)	251 (3.2)	0.011
Alcohol abuse (N [%])			
No (<8U/wk)	3542 (86.5)	6854 (87.3)	-0.024
Yes (>=8U/wk)	312 (7.6)	540 (6.9)	0.029
Unknown	243 (5.9)	461 (5.9)	0.003
Alcohol use (N [%])			
Never	1564 (38.2)	3295 (41.9)	-0.077
<1 drink/week	1055 (25.8)	1903 (24.2)	0.035
1-7 drinks/week	923 (22.5)	1656 (21.1)	0.035
>=8 drinks/week	312 (7.6)	540 (6.9)	0.029
Unknown	243 (5.9)	461 (5.9)	0.003

Type of site (N [%])			
General practice /primary care	246 (6.0)	401 (5.1)	0.039
Specialist office	1479 (36.1)	2413 (30.7)	0.114
Community hospital	1267 (30.9)	2659 (33.9)	-0.063
University hospital	993 (24.2)	2217 (28.2)	-0.091
Out-patient health care center	28 90.7)	68 (0.9)	-0.021
Anticoagulation clinics	19 (0.5)	24 (0.3)	0.026
Missing	8 (0.2)	39 (0.5)	-0.051
Transient ischaemic attack (N [%])	183 (4.5)	423 (5.4)	-0.042
Unknown	2 (0.0)	4 (0.1)	-0.001
Pulmonary embolism (N [%])	39 (1.0)	41 (0.5)	0.050
Unknown	59 (1.4)	84 (1.1)	0.033
Deep vein thrombosis (N [%])	60 (1.5)	90 (1.1)	0.028
Unknown	69 (1.7)	99 (1.3)	0.035
Angina pectoris (N [%])	280 (6.8)	631 (8.0)	-0.046
Unknown	55 (1.3)	53 (0.7)	0.067
Congestive heart failure (N [%])	783 (19.1)	1519 (19.3)	-0.006
Unknown	35 (0.9)	52 (0.7)	0.022
Rheumatic heart disease (N [%])	11 (0.3)	34 (0.4)	-0.028
Unknown	66 (1.60	63 (0.8)	0.074
Additional antiplatelets (N [%])	578 (14.1)	1012 (12.9)	0.036
Peripheral artery disease (N [%])	99 (2.4)	196 (2.5)	-0.005

Unknown	28 (0.7)	47 (0.6)	0.011
Cancer (N [%])	417 (10.2)	840 (10.7)	-0.017
Unknown	79 (1.9)	91 (1.2)	0.063
Hyperlipidemia (N [%])	1734 (42.3)	3249 (41.4)	0.019
Unknown	104 (2.5)	159 (2.0)	0.034
Hepatic disease (N [%])	41 (1.0)	77 (1.0)	0.002
Unknown	66 (1.6)	97 (1.2)	0.032
Non-central nervous system arterial embolism (N [%])	17 (0.4)	30 (0.4)	0.005
Unknown	16 (0.4)	22 (0.3)	0.019
Presence of complex aortic plaque (N [%])	26 (0.6)	61 (0.8)	-0.017
Unknown	57 (1.4)	131 (1.7)	-0.022
Proton Pump inhibitors (N [%])	1023 (25.0)	2022 (25.7)	-0.018
Chronic gastrointestinal diseases (N [%])	603 (14.7)	1042 (13.3)	0.042
Unknown	67 (1.6)	108 (1.4)	0.021
Vascular disease (N [%])	433 (10.6)	843 (10.7)	-0.005
Dementia (N [%])	25 (0.6)	51 (0.6)	-0.005
Misssing	70 (1.7)	86 (1.1)	0.052

CHA_2DS_2-VASc, congestive heart failure, hypertension, age ≥ 75 years, diabetes, stroke/transient ischaemic attack, vascular disease, age 65 to 74 years, sex category, HAS-BLED, hypertension, abnormal renal/liver function, stroke, bleeding history or predisposition, labile International Normalised Ratio, elderly, drugs or alcohol concomitantly, NOAC, non-

vitamin K antagonist oral anticoagulants, SD, standard deviation, VKA, vitamin K antagonists.

Medication usage predisposing to bleeding is defined as antiplatelet therapy or inhibitors of cyclooxygenase 2 or other non-steroidal anti-inflammatory drugs (NSAIDs).
